# A systematic review including meta-analysis of work environment and burnout symptoms

**DOI:** 10.1186/s12889-017-4153-7

**Published:** 2017-03-16

**Authors:** Gunnar Aronsson, Töres Theorell, Tom Grape, Anne Hammarström, Christer Hogstedt, Ina Marteinsdottir, Ingmar Skoog, Lil Träskman-Bendz, Charlotte Hall

**Affiliations:** 1 Department of Psychology, University of Stockholm, S-10691 Stockholm, Sweden; 2 Stress Research Institute, University of Stockholm, Stockholm, Sweden; 3 Department of Neuroscience, Karolinska Institutet, Stockholm, Sweden; 4 Health Care Centre, Norrtälje, Sweden; 5 Department of Public Health and Clinical Medicine, University of Umeå, Umeå, Sweden; 6 Division of Occupational Medicine, Institute for Environmental Medicine, Karolinska Institutet, Solna, Sweden; 7 Division of Psychiatry, University of Linköping, Linköping, Sweden; 8 Department of Psychiatry and Neurochemistry, University of Gothenburg, Gothenburg, Sweden; 9 Division of Psychiatry, University of Lund, Lund, Sweden; 10 Swedish Council of Health Technology Assessment, Stockholm, Sweden

**Keywords:** Burnout, Emotional exhaustion, Cynicism, Personal accomplishment, Job control, Job demands, Social support, Review, Meta-analyses, GRADE system

## Abstract

**Background:**

Practitioners and decision makers in the medical and insurance systems need knowledge on the relationship between work exposures and burnout. Many burnout studies – original as well as reviews - restricted their analyses to emotional exhaustion or did not report results on cynicism, personal accomplishment or global burnout. To meet this need we carried out this review and meta-analyses with the aim to provide systematically graded evidence for associations between working conditions and near-future development of burnout symptoms.

**Methods:**

A wide range of work exposure factors was screened. Inclusion criteria were: 1) Study performed in Europe, North America, Australia and New Zealand 1990–2013. 2) Prospective or comparable case control design. 3) Assessments of exposure (work) and outcome at baseline and at least once again during follow up 1–5 years later. Twenty-five articles met the predefined relevance and quality criteria. The GRADE-system with its 4-grade evidence scale was used.

**Results:**

Most of the 25 studies focused emotional exhaustion, fewer cynicism and still fewer personal accomplishment. Moderately strong evidence (grade 3) was concluded for the association between job control and reduced emotional exhaustion and between low workplace support and increased emotional exhaustion. Limited evidence (grade 2) was found for the associations between workplace justice, demands, high work load, low reward, low supervisor support, low co-worker support, job insecurity and change in emotional exhaustion. Cynicism was associated with most of these work factors. Reduced personal accomplishment was only associated with low reward. There were few prospective studies with sufficient quality on adverse chemical, biological and physical factors and burnout.

**Conclusion:**

While high levels of job support and workplace justice were protective for emotional exhaustion, high demands, low job control, high work load, low reward and job insecurity increased the risk for developing exhaustion. Our approach with a wide range of work exposure factors analysed in relation to the separate dimensions of burnout expanded the knowledge of associations, evidence as well as research needs. The potential of organizational interventions is illustrated by the findings that burnout symptoms are strongly influenced by structural factors such as job demands, support and the possibility to exert control.

**Electronic supplementary material:**

The online version of this article (doi:10.1186/s12889-017-4153-7) contains supplementary material, which is available to authorized users.

## Background

The first articles on burnout were published in 1974 by Herbert Freudenberger [1], a clinical psychologist working in an alternative health care agency, and in 1976 by Christina Maslach [2], a social psychologist studying workplace emotions. Since then the research field has grown extremely rapidly and according to an article 2014 in the journal Burnout Research there are more than 1000 journal articles on different aspects of burnout published every year [3].

The definition of burnout includes three dimensions; *emotional exhaustion*, *cynicism or depersonalization* and *reduced personal accomplishment* [4, 5]. Emotional exhaustion represents the basic individual stress dimensions of burnout and refers to feelings of being depleted of one’s emotional and physical resources. The cynicism (or depersonalization) component represents the interpersonal context dimension of burnout. Reduced accomplishment represents the self-evaluation dimension of burnout and refers to feelings of incompetence and a lack of achievement and productivity at work [5].

Burnout is seen as a process in time: increased coping efforts with external demands leads to emotional exhaustion, which is a trigger for depersonalization, which in turn leads to diminished personal accomplishment adding to further emotional exhaustion in a vicious cycle etc. [5]. This time-lag is important from a methodological perspective. All persons with high scores on emotional exhaustion have not reached depersonalisation and reduced personal accomplishment.

Burnout is generally based on self-reports. The original Maslach Burnout Inventory (MBI) is based on experiences of workers in helping professions in the 1970:s. Later research recognized that burnout is not a unique phenomenon in human service professions and therefore competing versions of the original Maslach Burnout Inventory, such as the MBI-General Survey, and other instruments for burnout screening were developed. Versions of the MBI are still the most frequently used questionnaires, but other well validated scales exist such as the Pines Burnout measure [6] and the Copenhagen Burnout Inventory [7].

The causes of burnout are generally divided into situational and individual ones, including personality. In the core of situational work environment factors there are different aspects of job demands, individual control at work and level of social support [8].

Psychologically, burnout is regarded as a continuous variable with individuals experiencing various levels of burnout. But because doctors and the occupational health services meet many people suffering from various symptoms of burnout, there are practical reasons for a differentiation between people who fulfil the pre-required criteria for burnout. This is especially relevant in Sweden, where in contrast to most other countries, burnout and exhaustion syndrome are accepted diagnoses and justify compensation.

Practitioners need a dichotomous concept especially when interacting with representatives of the social insurance and financial compensation systems in cases of sick leave or disability, as well as when evaluating the effects of treatment efforts. Both statistical and diagnostic criteria have been used to transform a continuous scale into a dichotomy that discriminates between “cases” and “non-cases” [9]. The current review relates to the practical context, namely to provide scientific evidence for possible associations between working conditions and near-future development of burnout. The review does not assess magnitudes of different associations between the dimensions. However, as none of the original studies included in the review used any “cut off points” for a dichotomization in burnout/no burnout in the medical and dichotomous sense, we have chosen to use the term *burnout symptoms* instead of burnout.

Despite these difficulties, estimations of burnout levels have been conducted in different populations. In Swedish working population studies the prevalence has varied between 6 and 18% [10, 11] and in the public debate in Sweden burnout is seen as a serious public health issue. Based on a large epidemiological study including around 12,000 Dutch employees, it is estimated that about 16% of the Dutch working population is at risk of burnout and that each year 6% of the Dutch workforce develop serious burnout complaints [12]. In a review of 17 studies of emergency nurses, who have been assumed to be vulnerable to burnout, on average more than 25% exceeded the cut-off for the three dimensions of burnout [13]. In a North-American context there is a Canadian study of a sample of 63 workplaces and 2162 employees [14]. The prevalence estimate of emotional exhaustion was 11,8, cynicism 8,1, professional efficacy 11,1 and total burnout 3,9. The researchers used MBI-GS.

Few of the systematic reviews and meta-analyses of burnout in a working context published during the last 15 years have required a longitudinal design; most are based on studies applying a cross-sectional design. There have been many meta-analyses of different occupational groups mostly based on studies applying cross-sectional design. A common conclusion from those occupational group studies is the need for more and methodologically better studies - which underlines the need for reviews of high quality studies.

The most recent systematic review [15] was based on six longitudinal studies. It was restricted to psychosocial working conditions and to the dimension of emotional exhaustion and a total burnout measure. The results from that review “point to a relationship between psychosocial working conditions and the development of emotional exhaustion/burnout. Particularly high job demands seem to play a role in the development of emotional exhaustion”. The influence of low job control on burnout dimensions was inconsistent.

Alarcon [16] reviewed studies from 1990 to 2010 and analysed 231 samples on the associations between job demands, resources and job attitudes with any of the three dimensions of MBI and excluded studies with total MBI measures of burnout and also other measures of burnout. Most of the primary studies included used cross-sectional designs, which Alarcon concludes as being an important limitation. The results “suggested that higher demands, lower resources, and lower adaptive organizational attitudes were associated with burnout and with all the three burnout dimensions. In particular, results in the study showed stronger relations than previous meta-analyses have suggested”.

The aim of this review was to provide systematically graded evidence in longitudinal studies for possible associations between working conditions and near-future development of symptoms of burnout, emotional exhaustion, cynicism and reduced personal accomplishment among the employees. Compared with most previous systematic reviews the present study had a broader approach, both regarding exposure factors and burnout dimensions, which have mostly been restricted to emotional exhaustion. In addition we have somewhat different criteria than most other studies for specifying the scientific evidence since we are using the GRADE-system.

## Methods

In accordance with our interest in generating knowledge for practitioners and decision makers in the medical and insurance systems we have chosen to use the internationally recognized GRADE- system for our scientific evaluation [17]. The GRADE-system has been developed with the purpose of grading evidence for intervention effects in health care, but the system has been adapted to epidemiological evaluation. The GRADE system is often applied in reviews conducted within the Cochrane Collaboration and is increasingly used internationally e.g., by the World Health Organization. Accordingly results from systematic reviews can be more easily compared. As far as the authors know there is no published study that has used the GRADE system for evaluating the evidence of burnout research.

We conducted and funded this systematic review within the framework of the Swedish Agency for Health Technology Assessment and Assessment of Social Services (SBU), a public agency with the charge of providing impartial and scientifically reliable information to decision makers and health care providers [18].

As far as it was possible the presented review was performed in accordance with PRISMA [19].

### Search strategy

The search strategy and inclusion criteria were formulated by the expert group, which was recruited among Swedish academic high ranking specialists in psychiatry (three), epidemiology and stress research (three), work psychology (one) and family practice (one).

**Fig. 1:**
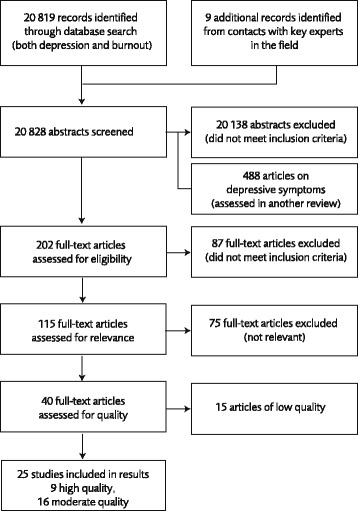
Flow chart of the literature search, screening, review- and quality assessment

Systematic literature search was performed for the period 1990 to 2013 (August) in the following data bases: PubMed, Embase, Psychinfo, Arbline (Swedish database), Cochrane library and NIOSHTIC-2. A combination of controlled search words (e.g. Medical Subject Headings/MeSH) and free-text words were used. The search strategy (exemplified by the search in the PubMed data base) is available at http://www.sbu.se/223E. The literature search, which also included a parallel study of depression articles, resulted in 20 819 abstracts. The depression study has been published [20]. Only original studies, not reviews, were used. Because some articles covered both outcomes – burnout and depression - it is not possible to give the exact number of burnout studies in the steps of the review process, see Fig. 1.

### Inclusion criteria

The inclusion criteria for studies were:

1. The study should have examined the importance of the work environment for symptoms of burnout. Our review was not confined to any specific kind of work environment factors. Psychosocial factors but also exposure to physical/chemical/ergonomic factors were screened (associations between those latter exposures and burnout have been investigated in cross-sectional studies so we wanted to explore if there could be studies of higher quality).
2. The studies should be relevant for Swedish conditions and focused on people at work. The review was confined to studies from the Western world (Europe, North America, Australia and New Zealand).
3. The studies should have been published between the years 1990 and 2013 in scientific journals and written in English. The time limitation was based on our assumption that the conditions in working life have changed so extensively during the last 25 years that the external validity and relevance of older studies may be questioned.
4. A minimum of 100 persons should have been included in the exposed group and the results were controlled for at least age and gender.
5. Only prospective cohort, case control (with design equivalent to prospective) and randomized intervention studies were included. By case control studies with “design equivalent to prospective” we are referring to studies with strict definition of cases recruited in a representative way in the same population as the control group.
6. Assessments of exposure should have been made before burnout onset.
7. Doublets were systematically identified and only the most relevant publication in a doublet was included.

### Analyses of relevance and quality

Abstract screening and full-text assessment for inclusion criteria were conducted by three persons: an information specialist at SBU, a psychiatrist and a specialist in occupational medicine. This first screening resulted in 115 full-text articles that met the inclusion criteria. Then the recruited experts judged relevance and quality of these 115 studies on the basis of the relevance/quality criteria, their experience as researchers and their knowledge of the field. The expert group was divided into pairs with as widely differing specialities in the pair as possible. Concordance in judgements of relevance and quality (see below) was trained. After the training sessions, each member of the pair did the assessments separately, and then discordances were discussed within the pair. If disagreement remained, another pair was asked to make an independent judgement. If that decision was in disagreement with the first group, we made a joint decision in the whole group of experts. The articles were randomly assigned to the four pairs (with avoidance of author bias).

In the first phase, the expert group judged relevance. Detailed relevance criteria are presented in http://www.sbu.se/upload/Publikationer/Content0/1/223E/Inclusion%20criteria_occupational%20exposure_depression_burnout.pdf.

Secondly, the quality assessments were performed. Three levels of quality rating were used, and in the final grading process only the two highest quality levels studies were accepted. Accordingly the important dividing line was between poor and medium high quality whereas the distinction between medium high and high was less crucial. Studies on the borderline between low and medium high quality were accordingly re-examined by the whole group. In all steps in the review procedure, pre-set evaluation forms were used. They were very detailed and aimed at focused attention to formulation of research question, selection of study population, recruitment, attrition, choice of exposure and outcome measures and statistical methods. The relevance form contained nine specified questions concerning study design, associations studied, research questions, population, follow up time. The quality form contained 31 questions and additionally five summarizing questions regarding selection, exposure assessment, outcome assessment, attrition and interpretation. For a more detailed description of the criteria and evaluation forms, see http://www.sbu.se/223E.

The following aspects of quality were considered:

1. Representativeness of study sample
  Studies based on population samples and samples from companies and occupational groups were accepted. Representativeness and ways of defining and recruiting the sample as well as attrition in different steps were considered. Statistical considerations and insightful discussions of possible consequences of a systematic drop-out for findings were required in case of marked drop-out problems.
2. Confounding. Age and gender should have been considered. Most of the included studies were based upon specific occupational cohorts, for instance dentists, nurses, primary school teachers. In these studies socioeconomic status was not considered to be confounding. In the studies based upon the general working population income, education or social class were used as confounders. Life habits, e.g. smoking habits and alcohol consumption, were not taken into account as potential confounders.
3. Prospective data collection. All results of the studies included in this review are based upon assessments of exposure at baseline and assessment of burnout at follow-up at least 1 years later. In the calculations of associations, a design with either exclusion of subjects with burnout at baseline or adjustment for baseline level of burnout was required.
4. For both exposure and outcome assessments psychometrically standardised and validated methods were required. Well established methods enable comparisons across studies and therefore contribute to higher quality rating.
5. Designs that enable the analysis of a dose response relationship contributed to a high quality rating. For instance, in a few studies the work environment was assessed in two or three subsequent waves and the development of symptoms of burnout followed up after the last exposure assessment. Exposure to defined work environment factors on one, two or three occasions could be regarded as a progressive duration of exposure and was regarded as equivalent of a dose-response analysis.

Even between studies of specific work environment factors there were differences with regard to operationalization of exposure. Examples are job strain (combination of high psychological demands and low decision latitude) and effort reward imbalance (combination of high effort and poor reward). Since the overall aim of the present study was to grade total evidence, not to assess magnitude of associations, and since it was impossible to re-construct operationalizations in such a way that they would match one another we decided to use the definitions presented by the authors themselves and to mostly abstain from assessment of overall magnitude of the different relationships.

The final list of studies judged to be of high or medium high quality is listed in Additional file 1.

### The evidence procedure

An important aspect of the systematic review process was to systematically and transparently assess the scientific evidence. According to the GRADE instructions explicit consideration should be given to each of the GRADE criteria for assessing the quality of evidence (risk of bias/study limitations, directness, consistency of results, precision, publication bias, magnitude of the effect, dose-response gradient, influence of residual plausible confounding and bias “antagonistic bias”) although different terminology may be used.

The GRADE-system uses four levels of evidence. For level 4 (=High), randomized trials are required and there were no such published relevant studies in our search. For observational studies of the kind included in the present review, the highest possible grade is Moderate = 3 if there is sufficient reason for an upgrading from the normal level for such studies of 2 (=Limited). Level 1 (=Very limited) corresponds to evidence based on case reports and case series or on reports with downgraded evidence from observational studies.

We allowed for upgrading the scientific evidence when there was strong coherence of results between studies - according to the most recent guidelines [21]. Accordingly when there were many published observational studies of medium high or high quality with homogenous results the evidence was graded on level 3. Such upgrading was done for two exposures, job control and low workplace support. Level 3 can also be used when there are relatively few studies if there are unanimous findings with high odds ratios (above 2.0). There were no such cases in our review.

### Meta analyses/Forest plots

**Fig. 2:**
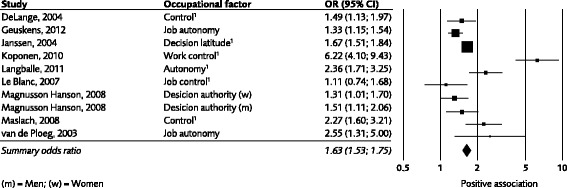
Association between low level of control (e.g. decision latitude and authority are used as synonyms of control) and development of emotional exhaustion. The graph is based on data from the least adjusted model in studies expressing the strength of the association either as odds ratios or as correlations (the latter have been transformed into odds ratios). ^1^Data have been re-calculated to show the association between low level of control and development of emotional exhaustion (data in these studies are originally presented as association between high level of control and emotional exhaustion)

In the studies reported results were reported as calculations of associations, e.g., expressed as odds ratios from multiple logistic regressions, multivariate correlations or multiple linear regression coefficients. As a base for the GRADE-assessment the results were transformed into multiple logistic regression odds ratios whenever possible. Forest plots were used for visual interpretation. To assist in illustrating the results, and as a contribution to the overall assessment, these forest plots (meta-analyses) were constructed when in at least two studies the same risk factor was analysed and mathematically comparable data was provided using the Comprehensive Meta- Analysis software package (www.meta-analysis.com/index.php). Since the participants in the various studies might be construed as coming from the same population (workers) or from different populations (i.e., according to each study’s inclusion criteria) we chose to use a fixed effects model. The strength of the scientific evidence, using data from all of the included studies (not just those illustrated in the meta-analyses), was determined by pairs of the authors of this paper and then discussed and confirmed by all authors. The forest plots were based on studies presenting associations as odds ratios, including studies where odds ratios could be calculated (see Fig. 2a–c legends). In forest plots, we chose to use data from the least adjusted model from each study. The main rationale for this was that these models were more comparable between studies than other models, since the more adjusted ones were adjusted to widely different potential confounders.

**Fig. 3:**
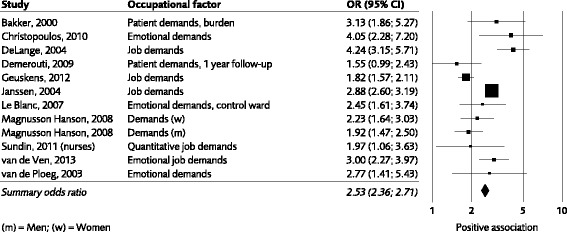
Association between job demands and development of emotional exhaustion. The graph is based on data from the least adjusted model in studies expressing the strength of the association either as odds ratios or as correlations (the latter have been transformed into odds ratios). Please note that data from two more studies (Lorente Prieto 2008 and Koponen, 2010) are included in the evidence-rated result; however data from these studies could not be illustrated in the graph due to the data format

Figure 2, 3, 4 shows forest plots for control, job demands and workplace support. For the other work factors studied, we present odds ratios and confidence intervals in the text and the number of studies which were used for the calculations of odds ratios.

**Fig. 4:**
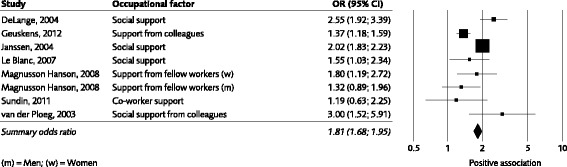
Association between low workplace support and development of emotional exhaustion. The graph is based on data from the least adjusted model in studies expressing the strength of the association either as odds ratios or as correlations (the latter have been transformed into odds ratios). Please note that data from two more studies (Burke 1995 and Koponen 2010) are included in the evidence-rated result; however data from these studies could not be illustrated in the graph due to the data format

The researchers in the included studies had chosen a wide range of different statistical measures to express associations between occupational exposure and burnout symptoms. Therefore, it was not possible to conduct formal mathematical homogeneity analyses including the entire data material. The expert group conducted a combination of mathematical and narrative sub-group analyses to explore whether the results were homogenous when subgroups of studies were compared. Comparisons concerned results from general population studies versus specific occupational cohorts, occupational environments in North America, Europe vs. the Nordic countries and for high vs medium high quality studies. Inspection of the confidence interval distributions, as well as estimated average odds ratios and their confidence intervals showed homogeneous results across those dichotomies. Homogeneity test could not be conducted on burnout screening instruments, since very few studies used any other instrument than the MBI [4]. Nor was it possible to carry out separate analyses for men and women, since the articles rarely presented gender specific results.

The numbers of participants in Table 1 are from the follow up. In a few studies such data were not available, and in those cases we present numbers of participants at the first data collection.

**Table 1:** A summary of the evidence for associations between work environment factors and different dimensions of burnout

Work factor	Burnout dimensions – participants, number of studies and scientific evidence											
	Emotional exhaustion			Depersonalisation, cynicism			Reduced personal accomplishment			^a^Burnout or symptoms of exhaustion		
Relationship between occupational environment and MORE burnout												
Low job control	19 769	9	⊕⊕⊕○	1 396	4	⊕⊕○○	123	1	⊕○○○	3 252	2	⊕⊕○○
Demands, unspecified	21 014	13	⊕⊕○○	1 354	4	⊕⊕○○	–	–	–	5 807	3	⊕⊕○○
Demands, emotional	1 591	5	⊕⊕○○	701	3	⊕⊕○○	123	1	⊕○○○	952	1	⊕○○○
Demands from patients	1 050	3	⊕⊕○○	1 040	3	⊕⊕○○	207	1	⊕○○○	–	–	–
Low co-worker support	12 788	4	⊕⊕○○	708	2	⊕⊕○○	123	1	⊕○○○	–	–	–
Low super-visor support	16 073	5	⊕⊕○○	708	1	⊕○○○	123	1	⊕○○○	952	1	⊕○○○
Low work-place support	19 747	9	⊕⊕⊕○	681	3	⊕⊕○○	485	2	⊕○○○	3 863	4	⊕⊕○○
High work load	2 290	7	⊕⊕○○	1 908	6	⊕⊕○○	821	3	⊕⊕○○	1 201	2	⊕⊕○○
Low reward	569	2	⊕⊕○○	569	2	⊕○○○	569	2	⊕○○○	–	–	–
Job insecurity	12 449	3	⊕⊕○○	–	–	–	–	–	–	–	–	–
Relationship between occupational environment and LESS burnout												
Workplace justice	921	3	⊕⊕○○	446	1	⊕○○○	446	1	⊕○○○	662	1	⊕○○○
The scientific evidence is insufficient												
Job strain	–	–	–	–	–	–	–		–	2 555	1	⊕○○○
Job development	–	–	–	–	–	–	–		–	952	1	⊕○○○
Work place conflicts	3 004	1	⊕○○○	–	–	–	–		–	–	–	–
Threats	585	1	⊕○○○	–		–	–		–	–	–	–
Lack of feedback	207	1	⊕○○○	–		–	–		–	–	–	–
Aspects of the occupational role	274	1	⊕○○○	274	1	⊕○○○	274	1	⊕○○○	952	1	⊕○○○
Long working week	523	1	⊕○○○	–	–	–	–	–	–	–	–	–
Physical environment	–	–	–	–		–	–	–	–	362	1	⊕○○○

^a^Includes outcome from Copenhagen Burnout Inventory (CBI), which measures symptoms of fatigue and exhaustion [7]

Criteria for evidence grading

⊕⊕⊕○There is moderate scientific evidence for a relationship between exposure and outcome. The result is based on studies of high quality

⊕⊕○○There is limited scientific evidence for a relationship between exposure and outcome. The result is based on studies of high or moderate quality

⊕○○○It is not possible to determine if there is a relationship between exposure and outcome. The motivation is that one or several conditions apply: 1) no study fulfilled the inclusion criteria, 2) none of the studies fulfilling the inclusion criteria were relevant to the hypothesis tested in the present review, 3) all relevant studies were of low quality or 4) studies were of high or moderate quality – but one or several limitations applied, e.g. inconsistency between studies

Has not been reviewed, due to lack of studies that fulfilled our inclusion criteria

Figure 1 is a summary of the number of articles that were perused in the different steps. As mentioned the project and search process also included depression as outcome for a review of depression and work environment [20]. As mentioned the first screening resulted in 115 full-text articles on organizational and psychosocial factors and symptoms of burnout that met the inclusion criteria and 40 of these were judged relevant by the expert group. Of these, 15 studies were of low quality in our three-graded scale of quality and were accordingly excluded. Finally, the grading of evidence were based on 25 articles of high or medium quality.

For physically demanding work, two studies met the criteria and for physical environment only one study was of medium or high quality. These three studies also included organizational and psychosocial factors, and are presented among the 25 studies on these factors. Concerning exposure to chemical and biological factors, noise and vibration, there were no studies meeting the inclusion criteria.

The datasets generated and/or analysed during the current study are available in the SBU repository, http://www.sbu.se/223E.

## Results

In all the 25 studies exposure was based on self-report in standardized and validated questionnaires. For job demand and control the most commonly applied instruments were the Job Content Questionnaire (JCQ) [22] and Copenhagen Psychosocial Questionnaire [23]. About ten different scales were used for the exposure assessments.

With regard to burnout, a handful of well validated instruments were used in the included articles. In 18 of the 25 studies, MBI, MBI-General Survey [4, 5] or the Dutch adaption of MBI, the Utrecht Burnout Questionnaire [24] were used. The other screening scales were the CBI (Copenhagen Burnout Inventory) [7], Oldenburg Burnout Questionnaire [25], Shirom-Melamed Burnout Questionnaire [26] and the Proactive Coping Inventory [27]. CBI was used in three studies and the other instruments in only one study each. In general there are high correlations between these measures. These were reasons for non-differential use of the measures.

### Work environment factors

Table 1 shows the results of the evidence grading process. The overall picture is that most of the studies were focused on emotional exhaustion and somewhat fewer on the dimension of depersonalisation. For (reduced) personal accomplishment and for the burnout measure there was often only one relevant study of sufficient quality in relation to a defined work factor. For the work factor low reward in relation to depersonalisation and to reduced personal accomplishment there were two studies of sufficient quality (Maslach & Leiter 2008; van der Ploeg & Kleber 2003), but the GRADE-assessment was “limited scientific evidence” because of inconsistent results in the two studies.

In the following, we present weighted odds ratios for the associations between the work factor and the burnout measure. For several of the exposure measures, such as job control, demands and workplace support, we also present forest plots.

Associations between *job control* and emotional exhaustion were analysed in a total of 9 studies (De Lange, Taris, Kompier, Houtman & Bongers, 2004; Geuskens, Koppes, van den Bossche & Joling, 2012; Janssen & Nijhuis 2004; Koponen, Laamanen, Simonsen-Rehn, Sundell, Brommels &Suominen, 2010; Langballe, Innstrand, Aasland & Falkum 2011; Le Blanc, Hox, Schaufeli, Taris & Peeters, 2007; Magnusson-Hansson, Theorell, Oxenstierna, Hyde & Westerlund, 2008; Maslach & Leiter 2008; Van der Ploeg & Kleber, 2003). The results are very consistent, eight of the nine point estimates were significantly above 1.0 (Fig. 2, note that some data were re-calculated from correlations to odds-ratios). The weighed odds ratio for these studies was 1.63, (95% CI 1.53 to 1.75). The results supported a moderate scientific evidence (grade 3) for a relationship between low job control and increased emotional exhaustion.

Two studies (Borritz, Bultman, Rugulies, Christensen, Villadsen & Kristensen 2005; Sundin, Soares, Grossi & Macassa, 2011b) used the burnout measure and provided support for limited scientific evidence for a relationship between low job control and increased burnout (grade 2).

*Job demands* were the most frequently studied occupational exposure in the included studies. Unspecified psychological demands were studied in relation to emotional exhaustion in 13 studies (Bakker, Schaufeli, Sixma, Bosweld & van Dierendonck, 2000: Chrisopoulos, Dollard, Winefield & Dormann, 2010; De Lange et al., 2004; Demerouti, Le Blanc, Bakker, Schaufeli & Hox, 2009; Geuskens et al., 2012; Janssen et al., 2004; Koponen et al., 2010; Le Blanc et al.,2007; Prieto, Salanova, Martinez & Schaufeli, 2008; Magnusson-Hansson et al., 2008; Sundin, Hochwalder & Lisspers, 2011a; Van de Ven, Van den Tooren & Vlerick., 2013; Van der Ploeg et al., 2003). For 11 of them (not Koponen et al., 2010 and Prieto et al., 2008) weighted odds ratios could be calculated (Fig. 3). The results were consistent, ten of eleven point estimates were significantly above 1.0 (weighted OR of 2.53, 95% CI 2.36 to 2.71).

Unspecified psychological demands were also studied in relation to depersonalisation and cynicism in 4 studies (Bakker et al., 2000; Demerouti et al., 2009; Le Blanc et al., 2007; Sundin et al. 2011a). For three of the four studies the point estimate was above 1.0 with a weighted odds ratio of 2.37, 95% CI 1.86 to 3.03).

Emotional demands had been studied in relation to emotional exhaustion in five studies (study Chrisopoulos et al., 2010; Le Blanc et al., 2007; Prieto et al., 2008; Van de Ven et al., 2013; Van der Ploeg et al., 2003). The point estimate was above 1.0 in the four studies where it could be calculated with a weighted odds ratio of 2.95, 95% CI 2.40 to 3.62.

Finally, demands from patients were studied in relation emotional exhaustion in three studies (Bakker et al., 2000; Demerouti et al., 2009; Sundin et al. 2011a). Two of the studies had 95% CI just below 1.0 (0.99-2.43 and 0.97 to 3.34) but the weighted odds ratio ended at 2.02 (1.50 to 2.72).

In summary, for all three aspects of demands (unspecified, emotional and from patients) the results supported limited scientific evidence in relation to emotional exhaustion and depersonalisation (grade 2). For demands and reduced personal accomplishment there were too few studies for any conclusion.

*Job strain*, i.e. the combination of high psychological demands and low decision latitude, was investigated in only one study (Ahola & Hakanen, 2007). The study indicated a strong association, but the scientific evidence was judged insufficient since the association with burnout was investigated only in one relevant study of sufficient quality.

Different sources of *social support* have been studied. The strongest evidence (grade 3) was found for *low workplace support and emotional exhaustion* without specified source (Burke & Greenglass 1995a;, De Lange et al., 2004; Geuskens et al., 2012; Janssen et al., 2004; Koponen et al., 2010; Le Blanc et al., 2007; Magnusson-Hansson et al., 2008; Sundin et al. 2011a; Van der Ploeg et al., 2003). The weighted odds ratio based on seven of these studies was 1.81 (95% CI 1.68 to 1.95) (Fig. 4). There was also evidence (grade 2) for an association between *low supervisor support* (Geuskens et al., 2012, Magnusson-Hansson et al., 2008; Sundin et al. 2011a; Theorell, Nyberg, Leineweber, Magnusson-Hansson & Westerlund 2012; Van der Ploeg et al., 2003), as well as for *low co*-*worker support* (Geuskens et al., 2012; Magnusson-Hansson et al., 2008; Sundin et al., 2011a; Van der Ploeg 2003) on one hand and emotional exhaustion on the other hand.

There were three studies on low workplace support and depersonalization (Burke et al. 1995a; Le Blanc et al. 2007; Sundin et al. 2011a) with a weighted ratio 1.59, (95% CI 1.11 to 2.26) and four studies (Borritz et al. 2005; Burke et al. 1995a,b; Sundin et al., 2011b) on low workplace support and burnout. The scientific evidence was rated limited (grade 2).

The *high work load* category refers to a mixed group of stressors all of which result in a large volume of work. In several of these studies the author refers to events leading to a high work volume. It differs from demands, which refer to rushed tempo (unspecified) or – and specified – emotional demands and demands from patients. There were 7 studies with focus on emotional exhaustion (Burke et al., 1995b; Demerouti et al., 2009; Langballe et al., 2011; Le Blanc et al., 2007; Prieto et al., 2008; Maslach et al., 2008; Van der Ploeg et al., 2003). A weighted odds ratio could be calculated for 5 of these which resulted in an odds ratio of 4.22 (CI % 3.50 to 5.11). There was also evidence for an association between high work load and the dimension of depersonalisation/cynicism. In six studies the outcome was described as depersonalisation. It was only possible to calculate weighted odds ratio for two of them (Maslach et al., 2008; Van der Ploeg et al., 2003) with an OR of 2.52 CI 95% 1.85 to 3.44). For two of the three studies on high work load and the outcome described as cynicism (Demerouti et al., 2009; Le Blanc et al., 2007) weighted odds ratios were calculated (3.03, CI 95% 3.03 (2.21 to 4.16). The analyses supported limited scientific evidence (grade 2) in relation to the dimension of depersonalisation/cynicism. There were three studies on work load and reduced personal accomplishment. A weighted odds ratio could not be calculated, but based on a narrative analysis the evidence was rated limited.

*Low reward* had a weighted odds ratio of 1.86 (1.37 to 2.52) in relation to emotional exhaustion (Maslach et al., 2008; Van der Ploeg et al., 2003).

*Job insecurity* had a weighted odds ratio of 1.39 (1.22 to 1.57) based on all three studies in relation to emotional exhaustion(Geuskens et al., 2012; Koponen et al., 2010; Magnusson-Hansson et al., 2008). The result for low reward and job insecurity supported limited scientific evidence (grade 2).

There was an association between *workplace justice* and decrease in emotional exhaustion (Koponen et al., 2010; Maslach et al., 2008; Ramarajan, Barsade & Burack, 2008) with an evidence grade of 2 (a weighted odds ratio of 0.35, 95% CI 0.27 to 0.45 based on the 3 studies).

Insufficient evidence (grade 1) was found for several factors, which are listed in the lower part of Table 1. Mostly there are no studies of sufficient quality. One study investigated the association between physical environmental factors (in a school environment, Burke et al., 1995a) and burnout and showed a significant association but one study is not enough for evidence. Finally, there were no relevant studies with sufficient quality of associations between exposures to vibrations, chemical and biological factors on one hand and burnout on the other hand.

## Discussion

The purpose of this systematic review was to describe, summarize and grade evidence for associations between work environment factors and the development of burnout in its three dimensions. To our knowledge, the current meta-analytic study and review is the first that has been carried out according to the GRADE system [17]. Compared with most previous systematic reviews the present study had a broader approach, both regarding exposure factors and the separate burnout dimensions, and somewhat different criteria compared to most other studies for specifying the scientific evidence through the use of the GRADE-system. Our review is based on a thorough literature search as well as upon a systematic evaluation of a large number of publications. Thus, it includes several environmental exposures, physical as well as psychosocial. Compared with the review and meta-analyses of Alarcon [16] our review includes more and newer articles and compared with the review of Seidler et al. [15] we included a much broader spectrum of work exposure factors as well as outcomes (all three dimensions of burnout).

Our focus on all three dimensions of burnout contributed to new knowledge concerning substantial associations as well as research needs. The literature search showed that there were many more studies of emotional exhaustion and work exposure factors as compared to depersonalization and reduced personal accomplishment. For personal accomplishment there was often only one study of sufficient quality. In most cases the result indicated a relationship between the investigated work factors and reduced personal accomplishment. Our focus on all three dimensions of burnout may be of interest in the ongoing discussion on the tendency to reduce burnout to exhaustion and to a clinical medical diagnosis. According to Maslach and Leitner the driving force in that development is not scientific progress but rather practical and administrative reasons within the ward and health system [28]. They argue instead for a development of research on burnout profiles based on all three dimensions and explorations of their probably different relations to different aspects of the work environment. Our study was not focused on burnout profiles. However, due to its focus on all the three dimensions of burnout and the broad spectra of work factors it may be a contribution to the development of the “burnout profile approach” and to expanded knowledge about the relation between the different burnout dimensions and different work environment aspects.

Our review confirms previous knowledge in support of the importance of control, demands and social support in the development of burnout. The scientific evidence was highest for associations between burnout symptoms and job control (moderate) and low workplace support (moderate). These work environment aspects have been central in many work environment empirical studies during the past three decades and therefore also in earlier meta-analyses. An interpretation is that job control “protects against” emotional exhaustion and burnout. Job control and workplace support comprise, together with job demands, the demand – control- support model [29, 30]. That model has been used in many studies of both cross-sectional and longitudinal design during the last decades. In general the model predicts mental ill-health [8, 31]. There was an indication of a relationship between the combination of high psychological demands and low decision latitude labelled job strain [29] and burnout, but the scientific evidence was judged insufficient since the association was supported by data from only one relevant study of sufficient quality.

The inclusion of several exposure factors, which have not been investigated in earlier systematic reviews and meta-analyses, contributed to new knowledge. For some of these factors (e.g. job strain, lack of feedback, job development, workplace conflicts and threats) there could be an indication of association with burnout or one of the dimensions of burnout, but since there was only one study of sufficient quality we judged it as insufficient evidence. One or two further studies on these aspects with results in the same direction will provide an improved basis for evidence grading.

For some of the other predetermined occupational factors, the extensive literature search did not identify any studies; alternatively the studies were of insufficient quality. There were few prospective studies with sufficient quality of the relationship between adverse chemical, biological and physical factors and burnout. A rationale for further research would be cross-sectional studies indicating associations. Alternatively a small number of published studies may be interpreted as low expectancy among researchers that these factors could be related to burnout.

The literature search included articles published up to August 2013. However, a more informal search in the scientific literature (PubMed and PsycInfo until June 2016) showed that few more prospective studies or other studies with relevance for the conclusions in our review have been published. A longitudinal study (9 years) by Evolahti, Hultell & Collins [32] show rather strong fluctuations in burnout level related to work aspects (control) and life situation among women, which may be interpreted as support for the importance of control. One relevant meta-analysis, largely based on cross-sectional studies, was identified. It showed that job control had a stronger relationship with depersonalization and personal accomplishment than with emotional exhaustion [33]. None of these studies would have changed our results and conclusions.

The present review has not specifically addressed the economic cost for societies related to burnout, but such costs may be considerable since burnout is related to performance, sickness absence and disability pension. A meta-analysis performed by Taris [34] supported the association between emotional exhaustion and performance. The evidence for the relationships between depersonalization, personal accomplishment, and performance was inconclusive. In a later meta-analytic study including 115 different studies Swider and Zimmerman [35], found that all three dimensions of job burnout had independent correlations with different aspects of costs and performance; .23 with absenteeism, .33 with turnover, and .36 with job performance.

### Methodological considerations

Our literature search was focused on the central databases for work environment factors and health. There may be a possibility that relevant studies could have been published in business and management focused journals, which were not included in our database search. Our experience is that advanced quantitative burnout researchers without exception publish their studies in psychological and medical journals. This was supported by a simple search in Business Source Premier on “Burnout” and “longitudinal” or “Burnout” and “prospective” and got about 30 articles1990 to 2016.

All the included 25 studies were based upon self-reports of occupational exposure and burnout outcomes. Until now, there are no large epidemiological prospective studies with more objective measures of the exposure and the outcome, i.e. not only self-reports but also assessments from professionals in the health care system. Data with subjective descriptions of both explanatory and dependent factors increase the risk of inflated associations [36]. This is particularly the case in cross-sectional studies while in prospective studies this risk is less pronounced. Accordingly, we only included prospective studies with data on initial symptoms and standardized measures of exposure and outcome. Future studies based on more objective assessment of exposure would be beneficial because an individual’s self-report of the work environment or how work is perceived may be influenced by the level of emotional exhaustion or burnout with obvious risk for reverse causation. Of course this does not mean that only “objectifiable” stress is harmful for health.

Unfortunately, the analytical structure only allowed us to look at single exposure factors. Of course, there may be interaction between different classes of exposures.

In the forest plots, we chose to use data from the least adjusted model from each study. The main rationale for this was that these models were more comparable between studies than other models, since the more adjusted ones were adjusted to widely different potential confounders. However, in general the differences between the least adjusted and the more adjusted models were rather small. For transparency, we have listed data in both least and most adjusted models, see tables at http://www.sbu.se/223E. An important point is that if a study presented data using several statistical models, all data from all models were included in the assessment of scientific evidence for all of the results presented in this systematic review.

As was mentioned, the expert group conducted sub-group analyses to explore whether the results were homogenous when subgroups of studies were compared. Accordingly results were compared for studies of participants from the general population vs. specific occupations, occupational environments in North America, Europe vs. the Nordic countries. Inspection of the confidence interval distributions, as well as sub-populations’ summary odds ratios and their confidence intervals showed homogeneity across those dichotomies.

Data from the 25 included studies did not allow for us to conduct statistically acceptable comparisons between men and women, since only few studies presented gender-separated data. A review by Purvanova and Muros [37] based on 183 studies, which included studies with cross-sectional design, showed that female employees were slightly more often emotionally exhausted than men, while men were somewhat more frequently depersonalized than women. Psychosocial factors outside work may interact with job exposure factors in relation to the risk of burnout, and such interactions may differ between men and women. This is a topic for further research with more advanced design and gender stratified analyses.

It cannot be excluded that some work environment factors may interact positively or negatively in relation to burnout outcomes. The lack of interaction shown in many cross-sectional studies does not exclude that such interactions exist because they may need a prolonged period of time to develop or did not have sufficient power for interaction tests. Our approach with focus on single factors can be explained by the lack of complex longitudinal studies where interaction effects had been studied. There is a need for future studies on this issue.

## Conclusions

While high levels of job support and workplace justice were protective for emotional exhaustion, high demands, low job control, high work load, low reward and job insecurity increased the risk for developing exhaustion. Despite the large number of studies on burnout, this systematic review found rather few studies of methodologically high quality. There is a need for more detailed studies of the different dimensions of burnout in relation to work exposure factors.

The potential importance of organizational interventions is illustrated by the findings that the development of the burnout syndrome is influenced by structural work environment factors such as job demands, low possibility to exert control and non-supportive workplaces. For most of the studied exposures the observed risks were of limited or moderate size, but despite this, the risks may still have a societal importance. If a work environment factor has a 30% prevalence and is associated with a relative risk of 1.6, the resulting population attributable risk is 7%, i.e. the proportion of disease in the exposed group that could be prevented by eliminating the risk factor. Accordingly, when an occupational exposure is frequent, even a moderately elevated risk associated with it becomes important in a societal context. Thus, the results presented here and elsewhere imply a strong need for improved psychosocial work environments to prevent burnout. The fact that the source of burnout often lies in structural work environment factors points at the potential value of multifactorial interventions. Strict studies of such interventions will generate more knowledge about the burnout phenomenon and would contribute useful knowledge for preventive measures.

## Additional file

Additional file 1: The 25 studies judged to be of high or medium high quality. (DOC 32 kb)
