# Supplementary material for: A systematic review including meta-analysis of work environment and burnout symptoms
Source: BMC Public Health. 2017 Mar 16;17:264. doi: 10.1186/s12889-017-4153-7 (PMC5356239; doi:10.1186/s12889-017-4153-7)
Supplement: Additional file 1: — The 25 studies judged to be of high or medium high quality. (DOC 32 kb) [file 12889_2017_4153_MOESM1_ESM.doc]

**Additional file 1**

**The 25 studies judged to be of high or medium high quality**

Ahola K, Hakanen J. Job strain, burnout, and depressive symptoms: A prospective study among dentists*. J Aff Disorders*. 2007;104(1):103-10.

Bakker AB, Schaufeli WB, Sixma HJ, Bosveld W, Van Dierendonck D. Patient demands, lack of reciprocity, and burnout: A five-year longitudinal study among general practitioners*. J Org Behav.* 2000;21(4):425-41. DOI: 10.1002/(SICI)1099-1379(200006)21:4<425::AID-JOB21>3.0.CO;2-#

Borritz M, Bültmann U, Rugulies R, Christensen KB, Villadsen E, Kristensen TS. Psychosocial work characteristics as predictors for burnout: findings from 3-year follow up of the PUMA Study. *J Occup Environ Med.* 2005;47(10):1015-25. [doi: 10.1097/01.jom.0000175155.50789.98](../../../../C:%5CUsers%5CGARON%5CDropbox%5CManus%20burnoutartikeln%5CJOVB%20manus%5Cinsända%20filer%20JOVB%2020%20juni%5CBlind%20Burnoutreview%20APA%20June%2029%20GA.docx)

Bourbonnais R, Brisson C, Vinet A, Vezina M, Abdous B, Gaudet M. Effectiveness of a participative intervention on psychosocial work factors to prevent mental health problems in a hospital setting. *Occup Environ Med*. 2006;63(5):335-42. doi:10.1136/oem.2004.018077

Burke RJ, Greenglass E. A longitudinal study of psychological burnout in teachers. *Hum Rel.* 1995;48(2):187-202. doi: 10.1177/001872679504800205

Burke RJ, Greenglass ER. A longitudinal examination of the Cherniss model of psychological burnout. *Soc Sci & Med*. 1995;40(10):1357-63. <http://dx.doi.org/10.1016/0277-9536(94)00267-W>

Chrisopoulos S, Dollard MF, Winefield AH, Dormann C. Increasing the probability of finding an interaction in work stress research: A two‐wave longitudinal test of the triple‐match principle. J *Occup Org Psychol*. 2010;1,83(1):17-37. doi: 10.1348/096317909X474173

De Lange AH, Taris TW, Kompier MA, Houtman IL, Bongers PM. The relationships between work characteristics and mental health: Examining normal, reversed and reciprocal relationships in a 4-wave study. *Work & Stress*. 2004;18(2):149-66. doi: 10.1080/02678370412331270860

Demerouti E, Le Blanc PM, Bakker AB, Schaufeli WB, Hox J. Present but sick: a three-wave study on job demands, presenteeism and burnout. *Career Dev Int*. 2009;14(1):50-68. <http://dx.doi.org/10.1108/13620430910933574>

Geuskens GA, Koppes LL, van den Bossche SN, Joling CI. Enterprise restructuring and the health of employees: a cohort study*. J Occup Environ Med*. 2012;54(1):4-9. doi: 10.1097/JOM.0b013e31823c766e

Hakanen JJ, Schaufeli WB, Ahola K. The Job Demands-Resources model: A three-year cross-lagged study of burnout, depression, commitment, and work engagement. *Work & Stress*. 2008;22(3):224-41. doi:10.1080/02678370802379432

Janssen N, Nijhuis FJ. Associations between positive changes in perceived work characteristics and changes in fatigue*. J Occup Environ Med*. 2004;46(8):866-75.

Koponen AM, Laamanen R, Simonsen-Rehn N, Sundell J, Brommels M, Suominen S. Psychosocial work environment and emotional exhaustion—Does a service provision model play a role?. Health policy*.* 2010;94(2):111-9. <http://dx.doi.org/10.1016/j.healthpol.2009.09.002>

Langballe EM, Innstrand ST, Aasland OG, Falkum E. The predictive value of individual factors, work‐related factors, and work–home interaction on burnout in female and male physicians: a longitudinal study. *Stress and Health.* 2011;27(1):73-87. doi: 10.1002/smi.1321

Le Blanc PM, Hox JJ, Schaufeli WB, Taris TW, Peeters MC. Take care! The evaluation of a team-based burnout intervention program for oncology care providers*. J Appl Psychol*. 2007;92(1):213.doi: 10.1037/0021-9010.92.1.213

Liljegren M, Ekberg K. The longitudinal relationship between job mobility, perceived organizational justice, and health. *BMC Public Health*. 2008;8(1):1. <http://www.biomedcentral.com/1471-2458/8/164>

Hanson LL, Theorell T, Oxenstierna G, Hyde M, Westerlund H. Demand, control and social climate as predictors of emotional exhaustion symptoms in working Swedish men and women. *Scand Pub Health. 2008*;36(7):737-43.doi: 10.1177/1403494808090164

Maslach C, Leiter MP. Early predictors of job burnout and engagement. *J Appl Psychol.* 2008;93(3):498. doi: 10.1037/0021-9010.93.3.498

Prieto LL, Soria MS, Martínez IM, Schaufeli W. Extension of the Job Demands-Resources model in the prediction of burnout and engagement among teachers over time. *Psicothema*. 2008;20(3):354-60.

Ramarajan L, Barsade SG, Burack OR. The influence of organizational respect on emotional exhaustion in the human services. *J Posit Psychol*. 2008;3(1):4-18.

Sundin L, Hochwälder J, Lisspers J. A longitudinal examination of generic and occupational specific job demands, and work-related social support associated with burnout among nurses in Sweden. *Work.* 2011;38(4):389-400.

Sundin Ö, Soares J, Grossi G, Macassa G. Burnout among foreign-born and native Swedish women: A longitudinal study. *Women & Health*. 2011;51(7):643-60. doi**:**10.1080/03630242.2011.618529

Theorell T, Nyberg A, Leineweber C, Hanson LL, Oxenstierna G, Westerlund H. Non-listening and self centered leadership–relationships to socioeconomic conditions and employee mental health. *PloS one*. 2012;24;7(9):e44119. doi: 10.1371/J.pone.0044119

Van de Ven B, van den Tooren M, Vlerick P. Emotional job resources and emotional support seeking as moderators of the relation between emotional job demands and emotional exhaustion: A two-wave panel study. *J Occup Health Psychol*. 2013;18(1):1. <http://psycnet.apa.org/doi/10.1037/a0030656>

Van Der Ploeg E, Kleber RJ. Acute and chronic job stressors among ambulance personnel: predictors of health symptoms. *Occup Environ Med*. 2003;1,60(suppl 1):i40-6. <http://dx.doi.org/10.1136%2Foem.60.suppl_1.i40>
